# Assessment of groundwater quality for human consumption and its health risks in the Middle Magdalena Valley, Colombia

**DOI:** 10.1038/s41598-024-61259-0

**Published:** 2024-05-18

**Authors:** Boris Lora-Ariza, Adriana Piña, Leonardo David Donado

**Affiliations:** HYDS-Hydrodynamics of the Natural Media Research Group, Universidad Nacional de Colombia, Sede Bogotá, 111321 Bogotá, Colombia

**Keywords:** Environmental monitoring, Geochemistry, Civil engineering

## Abstract

Groundwater is the primary source of water for people living in rural areas, especially during seasons when surface water is contaminated or unavailable. In Colombia, people use groundwater as drinking water without additional treatment. In addition, there is no infrastructure for wastewater collection and sewage treatment in the region of the Middle Magdalena Valley. The current study aims to evaluate the quality of groundwater in this region to determine any potential health hazards associated with its consumption. To reach the objective, three (3) physicochemical and microbiological sampling campaigns were carried out during different hydrological periods. A total of 428 groundwater samples were analyzed for over 28 parameters. The results were compared with the water quality standards proposed by the US EPA and Colombian regulations for human consumption. The analysis revealed the presence of total and fecal coliforms in 89% and 58% of the analyzed samples, respectively, identifying them as the main contaminants in groundwater. Furthermore, the pH levels did not meet the standards set by the US EPA in 33.8% of the cases and by Colombian regulations in 31.02%. Additionally, 32.8%, 17.6%, 14.3%, and 10.9% of the samples failed to meet the established thresholds for apparent color, magnesium, iron, and nitrates, respectively, under both standards. Moreover, only the analyses of selenium, mercury, and zinc complied with the quality standards under both regulatory frameworks. Based on the Colombian Drinking-Water Quality Risk Index (CDWQRI-IRCA), the risk associated with water quality meant for human consumption was assessed. The results showed that over 84% of the samples analyzed posed a high risk to human health, 4.6% posed a medium risk, 5.5% posed a low risk, and only 5.7% posed no risk at all. Additionally, official mortality statistics for children under four years old were reviewed, which revealed two deaths in 2019 due to Acute Diarrheal Disease (ADD) caused by consumption of contaminated water. Therefore, it is crucial to implement water treatment systems, establish aqueducts in rural areas, and conduct rigorous and systematic monitoring of drinking water to ensure it is safe for human consumption. It is also important to track morbidity and mortality rates associated with water consumption.

## Introduction

Groundwater constitutes the largest source of drinking water on the planet^1–3^ and the major water supply source in many regions^4^. In recent decades, groundwater use has shown a remarkable increase even in tropical countries, where the use of surface water sources has historically been prioritized^5–9^. The latter is primarily attributed to surface water contamination, global climate fluctuations, and land-use changes that modify river dynamics. Consequently, these changes adversely impact water availability to supply the increasing demand of a growing population and modifications in human settlement patterns^10–12^.

However, anthropic activities, such as human settlements, agricultural and industrial developments, have resulted in groundwater contamination due to the absence of wastewater treatment facilities and the presence of factors such as septic leakages, the utilization of fertilizers and pesticides, as well as the injection of fluids for storage or disposal purposes^6,10,13^. Additionally, groundwater can be affected by geogenic contaminants such as arsenic^14,15^.

Therefore, the deterioration of groundwater quality directly impacts public health, especially in regions where water supply infrastructure is scarce^16^. In such areas, water is often sourced from low-cost tube wells drilled by users with minimal technical specifications and without permission from environmental or local authorities^17^. As a result, there would not be control over either the quantity or the quality of the water that is consumed by the communities^18–20^.

The use of polluted water causes different diseases, especially in children^21,22^. The World Health Organization (WHO) defines Acute Diarrheal Disease (ADD) as a condition characterized by having three or more loose or liquid stools per day, along with symptoms such as nausea, vomiting, abdominal cramping, or malnutrition. ADD is considered the second major cause of death among children under 5 years of age, particularly in developing nations^23–26^. ADD is caused by the presence of coliforms and fecal coliforms in drinking water. Its occurrence is considered an indicator of water contamination and a risk for diseases^27–30^. In particular, *Escherichia coli*, a thermotolerant coliform, has the potential to cause diseases such as diarrhea, hemorrhagic colitis, hemolytic uremic syndrome, inflammatory colitis, dysentery, urinary tract infections, septicemia, and neonatal meningitis^31–34^. It is considered the most reliable biomarker of fecal contamination^30,31,35^. On the other hand, high levels of nitrate in groundwater can cause methemoglobinemia, cancer, arterial hypertension, immune system damage, and, above all, increase infant morbidity and mortality, considering that infants are the most susceptible to developing diseases associated with elevated nitrate concentrations in drinking water^36,37^.

Nevertheless, access to drinking water, proper sanitation, and hygienic practices are not guaranteed to all the population in developing countries such as Colombia. There, rural areas have inadequate coverage of water supply and wastewater collection systems, with less than 78% in 2019, and access to safe drinking water remained below 20% in 2014^38,39^. On the other hand, there are no groundwater quality monitoring stations or a systematic control of the quality of subsurface water resources, that is, there are no records that allow the evaluation of temporary variations in groundwater quality^40^.

In that context, the present work is an attempt to study the surface water and groundwater quality in the Middle Magdalena Valley (MMV) in Colombia and its implications for human health. The MMV is a rural region characterized by the lack of sanitation infrastructure, limited access of surface water during the dry hydrological period, recurrent flooding during the wet period, and intensive agricultural and industrial activities that have resulted in significant changes in land use over the past century. This region has transitioned from expansive livestock pastures to monocultures (single-crop farming) of rice, and more recently, to African palm^41^. There, groundwater has emerged as a reliable source of water for human consumption, livestock, agricultural and industrial requirements. Nonetheeless, the number of reported wells to the local environmental authorities (CAS, CSB and Corpocesar) is limited (8169 wells in 2021) but, a large number of wells still remain unreported, as observed during the field campaigns. The latter poses a huge number of wells that may not meet technical requirements, such as their well-head capture zones or proper seals on the surface. Further, in rural areas of Colombia, water for human consumption is often used directly from the water source without any treatment.

Therefore, a water quality assessment that allows the evaluation of human health risks due to groundwater contamination, is a challenging task that would be useful for stakeholders to identify the most common contaminants in the area and to propose proper management tools to improve the public health of the area.

In this paper, an assessment of the water quality for human consumption in rural areas of the Middle Magdalena Valley (MMV) in Colombia is presented. Analyses are based on results from three water sampling campaigns carried out during contrasting hydrological periods (wet and dry). Microbial contamination, physicochemical and trace concentrations of metals in water were measured. Reported values were contrasted with the US EPA Guidelines for Drinking-water Quality^42^ and Colombian regulations^43^. Finally, the risk associated with the quality of water intended for human consumption was evaluated using the Colombian Drinking-Water Quality Risk Index (CDWQRI-IRCA)^43^.

## Materials and methods

### Study area

**Figure 1:**
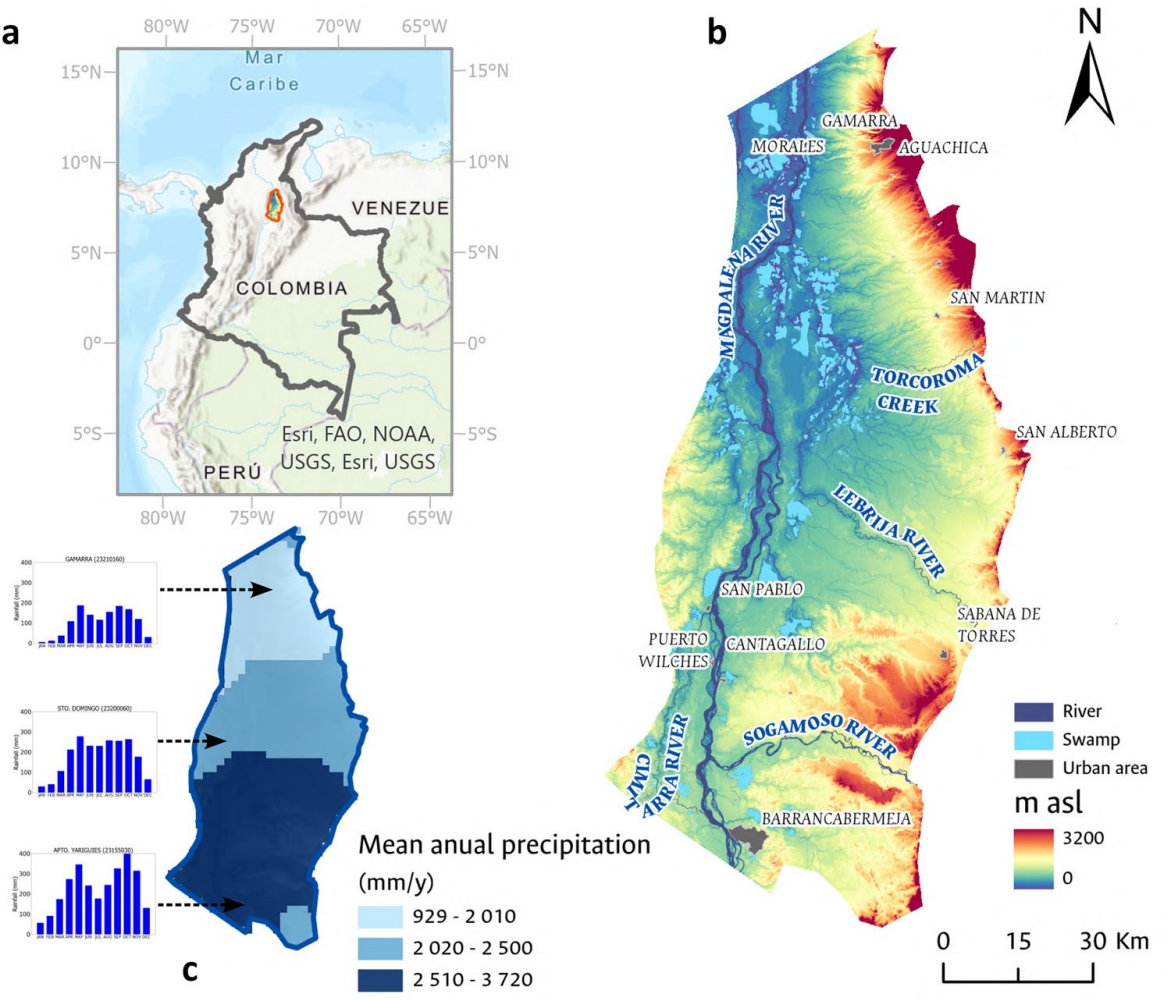
Study area showing (**a**) general location; (**b**) elevation in meters above mean sea level, municipalities, and major surfaces water bodies.This figure illustrates the spatial location of the MMV, with a particular focus on its topography. The HydroSHEDS DEM (World Wildlife Fund, 2006–2013), generated by NASA’s SRTM (Shuttle Radar Topography Mission) at a spatial resolution of 92 m, was employed for this purpose; (**c**) spatial distribution of mean annual precipitation. Additionally, this figure depicts the temporal distribution of precipitation evaluated at three meteorological stations managed by the Institute of Hydrology, Meteorology, and Environmental Studies of Colombia (IDEAM). Figures were generated using ArcGIS Pro software, Version 3.1 (https://pro.arcgis.com/es/pro-app/3.1/get-started/whats-new-in-arcgis-pro.htm).

The MMV is located in the northeastern region of Colombia, a plain zone with hills that do not exceed 288 masl (Fig. 1a). It has a surface area of 8500 km^2^ and is geographically situated between latitudes 8°30ʹ00″ N–6°54ʹ00″ N and longitudes 73°21ʹ00″ W–74°06ʹ00″ W. VMM is bounded by the piedmont of the Central and Eastern Ranges of the Andes (Fig. 1b).

The mean temperature is 27°. The rainfall regime has a bimodal behavior characterized by two rainy periods. The first one between March and May and, the second one between August and November. The annual average rainfall is about 2000 mm/y, showing a decreasing tendency from the southern (3700 mm/y) to the northern area (1000 mm/y)^40,44^ (Fig. 1c). The MMV is highly affected by the El Niño-Southern Oscillation (ENSO). During the wet season, floods affect communities and large areas of crops for long periods; in contrast, it is estimated a decrease of 60% in the run-off in the Magdalena-Cauca basin on a typical dry year^40,45,46^. This situation would be worse due to the increase in the frequency of extreme events associated with climate change. Even though approximately 4000 m^3^/s of water flows through the Magdalena River in the study area, it is classified as a poor water quality source based on the Water Quality Index (WQI)^47^.

The drainage network discharges from the South-East to the North-East direction in some swamps or in the Magdalena River (Fig. 1b). Historically, water supply for human consumption in the rural area of VMM has been sourced from surface water. However, in recent decades, there has been a shift in the water usage patterns associated with a reduction of surface water availability and water contamination. In the study area, it has been identified that only the municipalities of Aguachica, San Martín, and Barrancabermeja are equipped with wastewater treatment plants for the treatment of both domestic and non-domestic wastewater, thereby facilitating compliance with Colombian regulations for discharges^48,49^ There are no rural settlements with wastewater treatment systems. The disposal of domestic wastewater in rural settlements often occurs in public spaces, water bodies, or directly onto the soil, with excreta primarily managed through handmade pit latrines.

### Hydrogeological context

During the Mesozoic, the central and eastern Andes Mountain ranges were elevated, resulting in the formation of the MMV basin. It consists of sedimentary strata from the Cretaceous Period over a Jurassic igneous metamorphic basement with an average thickness of 8500 m^50–53^. To the south, there are outcrops of the Real Group, a Neogene formation whose thickness varies from 450 to 3500 m. In the north, there are Quaternary deposits outcrops underlying Real Group. Quaternary deposits have been categorized into eight geoforms, including alluvial fans and terrace deposits in the northeast, and others influenced by the deposition processes of the Magdalena River, Lebrija River, Sogamoso River, and primarily swamp zones, such as fluviolacustrine deposits, alluvial terraces, channel fluvial deposits, alluviums, terrace and deltaic cone deposits, and floodplain deposits^53,54^.

At the regional scale, groundwater flow is governed by the topographic effects^55,56^, following in the South-North direction discharging in the swamps system of the Magdalena river. Moreover, there are secondary flows in the East–West direction, coming from the Eastern range and discharging into the Magdalena River.

### Water sampling

**Figure 2:**
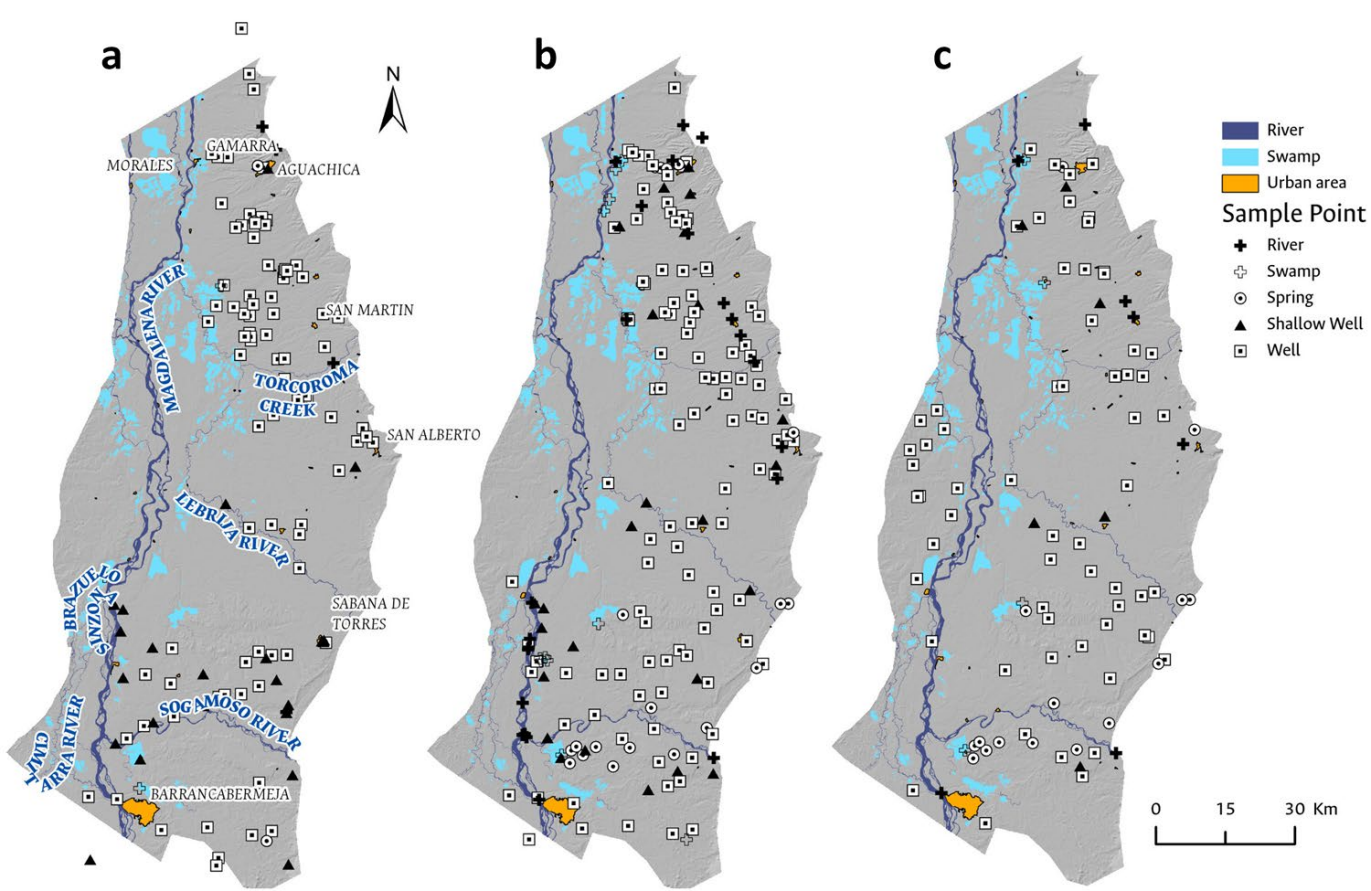
Map showing groundwater sampling sites during the (**a**) dry period in March 2020; (**b**) rainy period in November 2020; and (**c**) dry period in March 2021.These are categorized by water source type, including springs, shallow wells, wells, rivers, and swamps.

88 surface water samples and 428 groundwater samples were collected in three (3) field sampling campaigns for the analysis of physicochemical and microbiological parameters. The first campaign was conducted during the dry period in February 2020 (Fig. 2a); the second was carried out in November 2020 during the wettest period of the hydrological (Fig. 2b) year and, the third one in March 2021 at the end of the dry period (Fig. 2c). 2020 was cataloged as the La Niña phenomenon year, characterized by the increase in precipitation events.

The selection of groundwater sampling points was based on five criteria: (1) Spatial Distribution: Groundwater points were selected to ensure representativeness in the Neogene-Quaternary aquifer units outcropping in the study area, across different land uses/land covers, and within the main settlements of the study area. The selection of points less than 1 km apart was avoided to enhance spatial distribution. (2) Depth Distribution: Wells with depths greater than 10 m were chosen to ensure representation across the various aquifer units. (3) Accessibility: Selected points are within 1 km of the Colombian road network (primary, secondary, or tertiary) to minimize logistical issues and ensure sample integrity. However, this represents a limitation in the analysis of samples in the most remote areas, which may face greater challenges in water availability for human consumption. (4) Security: Wells in areas with public order issues, particularly on the eastern bank of the Magdalena River in the southern part of Bolivar department, were excluded. (5) Infrastructure: Sampled wells were equipped with a pumping system to adequately purge the well. Preference was given to wells with a known design, although it was not always possible to determine the depths of the filter intervals. For surface waters, sampling was carried out in the main rivers, creeks, and swamps throughout the study area to facilitate comparative analyses with groundwater.

A total of 190 groundwater sampling points were chosen. Those were classified as "shallow wells" with depths less than 30 m, "wells" with depths between 30 and 80 m, “deep wells” with depths greater than 80 m, and “springs”. In each campaign, an attempt was made to sample all the selected points, however, it was not possible due to access permissions; the second campaign was the most efficient with 170 points sampled.

Sampling procedures followed the established Colombian standards^57,58^. Wells were purged until the in-situ measured parameters (pH, Temperature, and Electrical Conductivity) stabilized. Those were measured using multiparametric probes (YSI ProQuatro and Schott handylab LF11) previously calibrated. 2 L plastic bottles were used for physicochemical analyses, 1 L amber glass bottle for microbiological analyses, and 0.5 L clear glass bottles for fats and oils analyses. Samples were preserved with nitric acid and thiosulfates as appropriate and all were stored at 4 °C.

### Laboratory analysis

The physicochemical and microbiological analyses were carried out in accordance with international standards at the Chemical and Environmental Engineering Laboratory of the Universidad Nacional de Colombia^57^. On the one hand, Temperature (Method SM 2550 B), Electrical Conductivity (Method SM 2510 B), pH (Method SM 4500 H + B), were measured both in-situ and in the laboratory. Meanwhile, Arsenic (SM 3111D), Barium (SM 3111D), Cyanide (proprietary method), Zinc (SM 2130 B), Chlorides (SM 4110B), Total Coliforms (SM 9222-H), Fecal Coliforms (SM 9222-H), Mercury (SM 3112 B), Nitrate (SM 4110B), Nitrite (SM 4110B), Ammonia Nitrogen (SM 4500 N), Silver (SM 3111 B), Lead (SM 3111 B), Selenium (3111 B), Sulfates (SM 4110B) were analyzed at the laboratory.

### Statistical analysis

Statistical analyses were performed using Python-Stats package. Basic statistics (mean, standard deviation, minimum and maximum values) were estimated for each parameter. The Kruskal–Wallis non-parametric statistical test was used to evaluate significant differences among samples, sampling period, and location^59,60^. This test enables the comparison of more than two independent samples, whether they have the same or different sample sizes, and mathematically, it is expressed as^59,61^:

1 $$H=\left(N-1\right)\frac{{\sum }_{i=1}^{g}{({r}_{i}^{*}-{r}^{*})}^{2}}{{\sum }_{i=1}^{g}{\sum }_{j=1}^{{n}_{i}}{({r}_{ij}-{r}^{*})}^{2}}$$

where $N$ corresponds to the total number of observations across all groups, $g$ is the number of groups, ${n}_{i}$ is the number of observations in group $i$, ${r}_{ij}$ represents the rank of observation $j$ from group $i$, ${r}_{i}^{*}=\frac{{\sum }_{j=1}^{{n}_{i}}{r}_{ij}}{{n}_{i}}$ is the average rank of all observations in group $i$, and ${r}^{*}=\frac{1}{2}(N+1)$ is the average of all the ${r}_{ij}$ values^61^.

### Water quality evaluation and spatial analyses

Water quality assessment followed the Colombian regulations^43^ and US EPA standards for human consumption^42^ as most people take water directly from wells with no treatment. Nevertheless, as Colombian regulation is less restrictive, the results shown here refer to US EPA standards. As a result, the portion of water samples above the allowed limits per parameter was quantified and identified along the study area. Their spatial behavior was compared with the land-use cover maps (1:100,000)^62^ and with the location of the most crowded settlements.

The Land Cover geographic object for the period 2018 provided by the Agustin Codazzi Geographic Institute (IGAC) at a scale of 1:100,000^62^ was interpreted by means of Landsat 8 remote sensing data. Based on the experience and scope of the research, the 36 land cover classes proposed by IGAC for the study area were reclassified into 5 categories: (1) agriculture, (2) urban fabric, (3) water bodies, (4) pastures, and (5) forests. From this classification, the land use/land cover type was determined at each sampled point.

### Human health considerations

The occurrence of total and fecal coliforms in water intended for human consumption has the potential to produce ADD, which is considered the leading cause of death in children under 4 years of age in developing countries^21^. For this reason, this research analyzed under-four children mortality caused by ADD during the year 2019, based on the information reported by the National Institute of Health of Colombia through the Public Health Surveillance System (SIVIGILA) for municipalities located in the study area^63^. Municipalities with mortality in children under four years of age were identified and related to the presence of pathogens in water supply points within their jurisdiction.

In the MMV, some settlements take groundwater for their water supply systems but lack drinking water treatment. The same occurs in the farms, where groundwater supplies agricultural activities and human consumption.

As the healthcare system in the rural area of VMM is precarious or non-existent, cases of ADD are often referred to medical centers in nearby major cities, some of which are even located outside the study area. Centralized infant mortality data are based on reports from clinics and hospitals, not on municipal reports, which could introduce biases in the information. Therefore, a risk analysis of water consumption in the Middle Magdalena Valley was conducted based on the laboratory analyses.

### Colombian Drinking-Water Quality Risk Index (CDWQRI-IRCA)

One method for assessing the risk related to the quality of water designated for human consumption involves the calculation of the Water Quality Indices (WQI)^64–66^. These indices enhance the understanding of water quality and serve as a tool for decision-making in the absence of specialized knowledge in specific fields related to water resources^67^. In this research, the Colombian Drinking-Water Quality Risk Index (CDWQRI), known in Colombia as IRCA, was estimated. This index is an arithmetic measure that relates the sum of weighted values, known as risk scores, for each of the parameters considered in the analysis, which do not meet the standards proposed by Colombian regulations^43^, to the total weighted values of all measured parameters, including those that meet the regulations. The CDWQRI-IRCA for each analyzed sample is determined by the Eq. (2)

2 $$CDWQRI-IRCA=\frac{\sum RSUAP}{\sum RSMP}$$

where *RSUAP* represents the risk score assigned to unacceptable parameters, while *RSMP* corresponds to the risk score of measured parameters. The weighted values are presented in the Table 1.

**Table 1:** CDWQRI-IRCA risk score.

Parameter	symbol	Risk score
Apparent color	*AC*	6.0
Turbidity	*T*	15.0
pH	*pH*	1.5
Free residual chlorine	${Cl}_{2}$	15.0
Total alkalinity	*Alk*	1.0
Calcium	*Ca*	1.0
Phosphates	${PO}_{4}^{3-}$	1.0
Manganese	*Mn*	1.0
Molybdenum	*Mo*	1.0
Magnesium	*Mg*	1.0
Zinc	*Zn*	1.0
Total hardness	*TH*	1.0
Sulfates	${SO}_{4}^{2-}$	1.0
Total iron	*Fe*	1.5
Chlorides	${Cl}^{-}$	1.0
Nitrates	${NO}_{3}^{-}$	1.0
Nitrites	${NO}_{2}^{-}$	3.0
Aluminium	*Al*	3.0
Fluorides	${Fl}^{-}$	1.0
Total organic carbon	*TOC*	3.0
Total coliforms	*TC*	15.0
Fecal coliforms	*FC*	25.0

Risk scores associated with each physicochemical and microbiological parameter used to estimate the CDWQRI-IRCA are presented^43^.

The risk level for human health evaluated using the CDWQRI-IRCA is categorized into five classes: Unhealthy, High, Medium, Low, and without risk. Higher values are associated with water unfit for human consumption, while values close to zero are considered suitable for human consumption Table 2.

**Table 2:** Classification of water according the CDWQRI-IRCA^43^.

CDWQRI-IRCA (%)	Risk classification
80.1–100.0	Unhealthy
35.1–80.0	Hight
14.1–35.0	Medium
5.1–14.0	Low
0.0–5.0	No risk

## Results and discussion

### Water quality in the MMV

The Kruskal and Wallis non-parametric test^59,60^ revealed statistical differences among the sampling period (wet or dry) and the location of the water sample (referred to the northern and the southern area) with p-values below 0.05 for Ca, Mg, ${HCO}_{3}^{-}$ and *Cl*^−^. Accordingly, most results are presented by sampling period. In Table 3, mean, minimum, maximum, and standard deviation values associated with both the physicochemical and microbiological analyses are presented for each sampling period, and Table 4 displays an overall statistical analysis for all the analyzed samples (Supplementary Information).

**Table 3:** Descriptive statistics on the 28 physicochemical and microbiological parameters of the water quality analyzed in the MMV.

Analisys			Dry period					Wet period				
Parameter	Symbol	Units	Number of samples	Mean	Min.	Max.	S.D.	Number of samples	Mean	Min.	Max.	S.D.
Arsenic	*As*	mg/L	230	0.033	0.001	5.819	0.385	196	0.003	0.002	0.019	0.002
Calcium	*Ca*	mg/L	234	15.519	0.500	221.000	19.698	220	15.805	1.000	100.000	14.387
Alkalinity	*Alk*	mg/L	234	91.143	0.500	593.000	79.436	223	91.946	0.000	452.000	79.012
Aluminum	*Al*	mg/L	230	0.666	0.005	133.000	8.746	201	0.128	0.097	2.080	0.168
Total Hardness	*TH*	mg/L	234	59.269	0.005	623.000	64.615	223	62.946	0.001	338.000	55.250
Iron	*Fe*	mg/L	234	0.295	0.100	17.000	1.182	219	0.205	0.100	2.700	0.316
Magnesium	*Mg*	mg/L	234	4.957	0.003	42.000	5.299	223	5.615	0.000	47.000	5.999
Manganese	*Mn*	mg/L	234	0.348	0.025	46.000	3.097	220	0.154	0.025	2.300	0.217
Barium	*Ba*	mg/L	230	0.361	0.003	0.833	0.134	196	0.389	0.002	0.586	0.208
Cadmium	*Cd*	mg/L	230	0.002	0.002	0.003	0.001	199	0.198	0.003	24.000	1.719
Cyanide	*CN*	mg/L	233	0.075	0.050	5.800	0.376	220	0.105	0.050	3.600	0.302
Zinc	*Zn*	mg/L	230	0.058	0.025	0.907	0.086	195	0.034	0.003	1.600	0.119
Chlorides	${Cl}^{-}$	mg/L	233	9.731	0.100	727.400	49.240	222	7.348	0.050	288.400	21.580
Copper	*Cu*	mg/L	230	71.775	0.025	16,000.000	1053.081	202	27.251	0.039	5400.000	378.977
Fecal coliforms	*FC*	MPN/100 mL	212	1705.245	9.000	160,000.000	11,247.100	213	2700.324	9.000	160,000.000	15,639.243
Total coliforms	*TC*	MPN/100 mL	211	17,544.081	9.000	420,000.000	53,295.619	212	8750.383	9.000	160,000.000	23,718.325
Apparent color	*AC*	PCU	234	21.670	4.000	1250.000	83.508	222	27.648	5.000	198.000	29.964
Chromium	*Cr*	mg/L	228	0.080	0.003	6.000	0.472	194	0.152	0.050	16.000	1.158
Fluorides	${Fl}^{-}$	mg/L	234	0.208	0.001	2.920	0.333	222	0.204	0.010	3.000	0.373
Mercury	*Hg*	mg/L	230	0.001	0.001	0.001	0.000	211	0.001	0.001	0.001	0.000
Nitrate	${NO}_{3}^{-}$	mg/L	234	4.334	0.005	63.700	9.415	222	4.359	0.100	73.400	10.727
Nitrite	${NO}_{2}^{-}$	mg/L	234	0.076	0.050	0.400	0.033	221	0.105	0.100	1.000	0.061
Nickel	*Ni*	mg/L	230	0.045	0.001	3.244	0.328	200	0.001	0.001	0.100	0.007
Lead	*Pb*	mg/L	230	0.045	0.010	4.532	0.375	197	0.037	0.010	2.200	0.202
Selenium	*Se*	mg/L	230	0.002	0.002	0.002	0.000	192	0.002	0.002	0.002	0.000
Sulfate	${SO}_{4}^{2-}$	mg/L	234	10.254	0.050	561.200	40.350	223	11.691	0.100	548.400	51.542
pH	*pH*	pH units	265	6.905	4.170	8.830	0.987	224	6.525	3.570	8.650	1.075
Silver	*Ag*	mg/L	230	0.058	0.025	5.114	0.334	197	0.009	0.001	0.050	0.004

These are presented according to the hydrological period in which the sampling was conducted.

**Table 4:** Descriptive statistics of the physicochemical and microbiological analyses without discriminating the hydrological sampling period.

Parameter	Units	Number of samples	Mean	Min.	Max.	S.D.
Arsenic	mg/L	426	0.019	0.001	5819.000	0.284
Calcium	mg/L	454	15,658.000	0.500	221,000.000	17,330.000
Alkalinity	mg/L	457	91,535.000	0.000	593,000.000	79,230.000
Aluminum	mg/L	431	0.415	0.005	133,000.000	6395.000
Total Hardness	mg/L	457	61,063.000	0.001	623,000.000	60,255.000
Iron	mg/L	453	0.251	0.100	17,000.000	0.879
Magnesium	mg/L	457	5278.000	0.000	47,000.000	5661.000
Manganese	mg/L	454	0.254	0.025	46,000.000	2231.000
Barium	mg/L	426	0.374	0.002	0.833	0.173
Cadmium	mg/L	429	0.093	0.002	24,000.000	1175.000
Cyanide	mg/L	453	0.089	0.050	5800.000	0.342
Zinc	mg/L	425	0.047	0.003	1600.000	0.103
Chlorides	mg/L	455	8568.000	0.050	727,400.000	38,344.000
Copper	mg/L	432	50,956.000	0.025	16,000,000.000	811,221.000
Fecal coliforms	MPN/100 mL	425	2204.000	9.000	160,000.000	13,635.500
Total coliforms	MPN/100 mL	423	13,136.800	9.000	420,000.000	41,450.300
Apparent color	PCU	456	24,580.000	4000.000	1,250,000.000	63,439.000
Chromium	mg/L	422	0.113	0.003	16,000.000	0.859
Fluorides	mg/L	456	0.206	0.001	3000.000	0.353
Mercury	mg/L	441	0.001	0.001	0.001	0.000
Nitrate	mg/L	456	4346.000	0.005	73,400.000	10,075.000
Nitrite	mg/L	455	0.090	0.050	1000.000	0.050
Nickel	mg/L	430	0.025	0.001	3244.000	0.241
Lead	mg/L	427	0.041	0.010	4532.000	0.308
Selenium	mg/L	422	0.002	0.002	0.002	0.000
Sulfate	mg/L	457	10,955.000	0.050	561,200.000	46,157.000
pH	pH units	489	6731.000	3570.000	8830.000	1046.000
Silver	mg/L	427	0.036	0.001	5114.000	0.247

**Figure 3:**
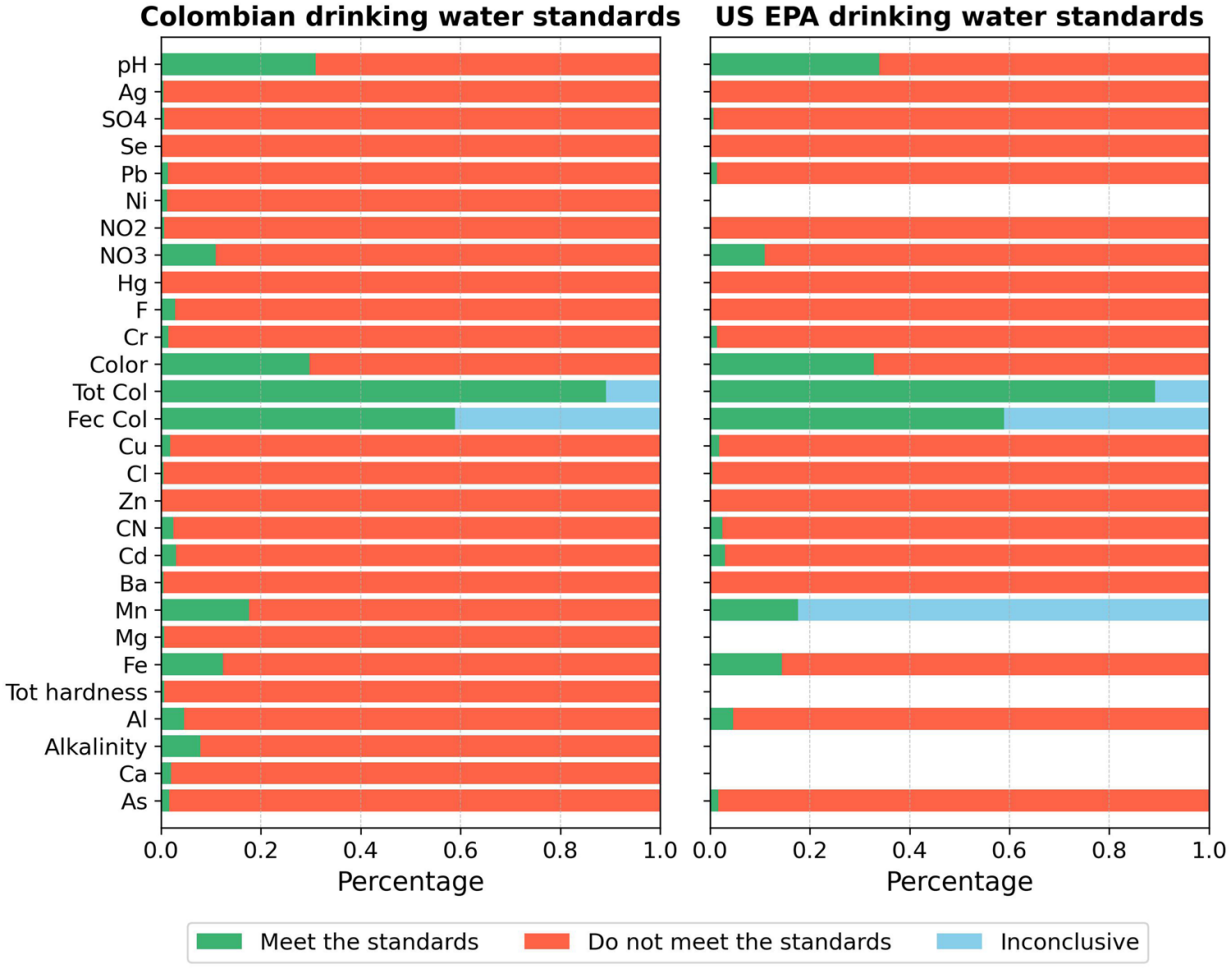
Comparison of water quality analyses between the standards proposed by the US-EPA and the Colombian Regulations for Drinking Water. Green bars represent the percentage of analyzed samples that meet the quality standards for human consumption. Orange bars indicate the percentage of samples that fail to meet the standards. Blue bars correspond to the percentage of samples from which no conclusions can be drawn.

The percentage of water samples that either comply or do not comply with the US EPA standards^68,69^ and Colombian regulation^43,49^ for human consumption is depicted in the Fig. 3. Among the 28 parameters evaluated, the Colombian regulations are found to be more stringent than the EPA standards for 10 specific parameters (Ba, Cyanide, Zn, Cu, Cr, *F*^−^, Hg, ${NO}_{2}^{-}$, Pb, and Se). Conversely, for three parameters (Mn, Cd, and pH), the Colombian standards are less stringent. Fecal coliforms and Total coliforms exceed the quality limits in 58% and 89% of the analyzed samples, respectively, in relation to both regulations. pH, color, Fe, Mn, ${NO}_{3}^{-}$, Al, Cs, Al, Cu, As, Pb, and ${SO}_{4}^{2-}$ parameters do not meet the quality standard in some percentage of sampled sites, while Ag, Se, Hg, *F*^−^, Zn, and Ba remain within the permissible values across the entire study area. Ni, Mg, Total Hardness, Alkalinity, and Ca were analyzed solely according to Colombian standards, as the EPA does not establish quality limits for these parameters in relation to human consumption.

**Figure 4:**
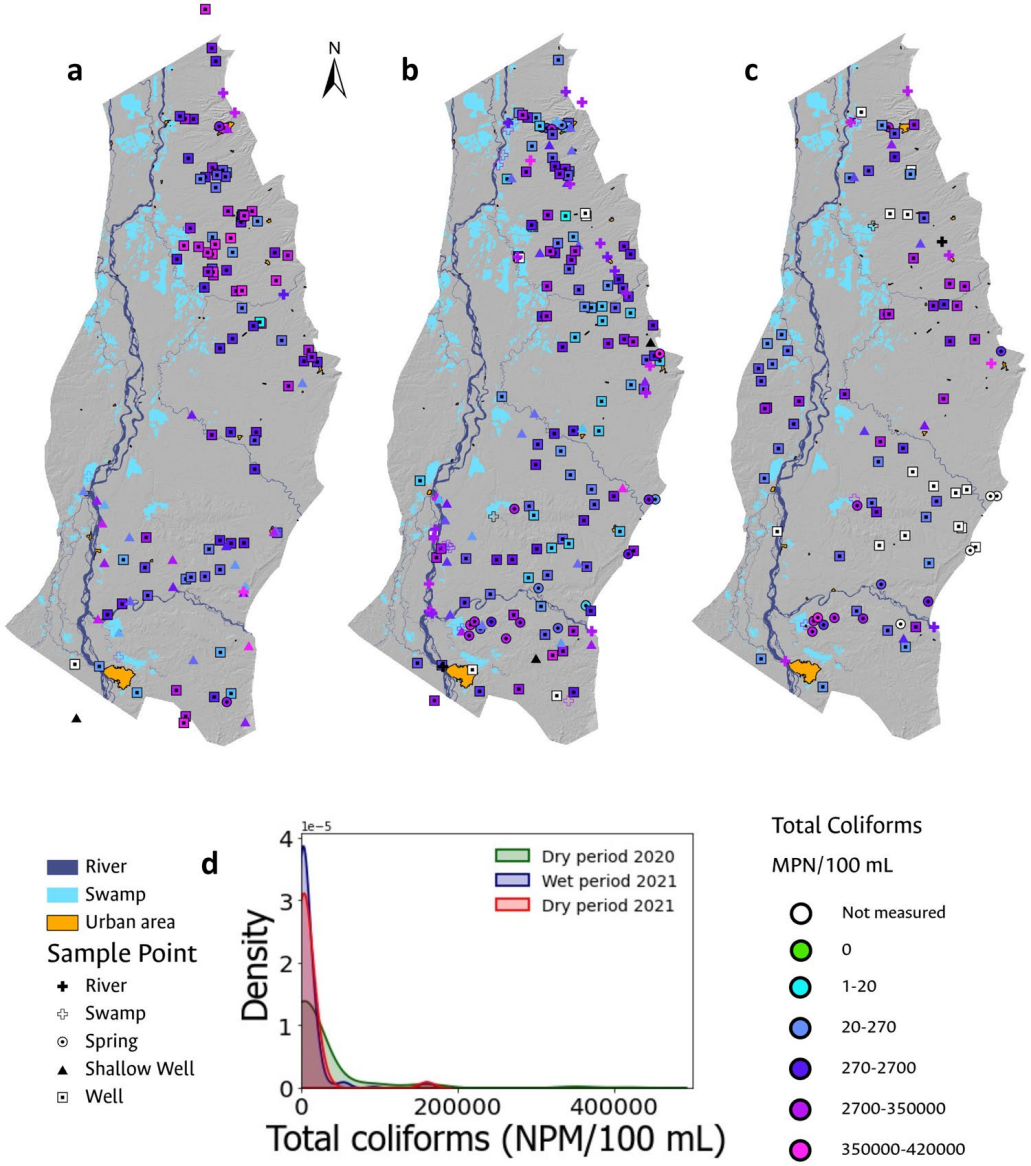
Spatial distribution of total coliform occurrence during (**a**) the dry period of 2020; (**b**) the rainy period of 2021; and (**c**) the dry period of 2021. Additionally, histograms with smoothed function fitting for the total coliform analyses evaluated in each field campaign are presented in (**d**).

Overall, total coliform occurrence is observed throughout the study area in the majority of the sampled sites (at least 89% of the analyzed samples), with higher concentrations near major population centers, except in the vicinity of Barrancabermeja, a major city with a robust sewer system in its urban area (Fig. 4).The most significant concentrations of pollution attributable to the presence of total coliforms were observed in the surface water samples under analysis, a finding consistent with expectations given that both domestic and industrial wastewater discharges are typically released into water bodies without prior treatment. Among the various surface water sites monitored, the Quebrada Cayumbita, situated in the southern region of the Sabana de Torres municipality, exhibited the highest level of total coliform contamination, registering 420,000 MPN/100 mL. In contrast, groundwater samples revealed the greatest total coliform concentration in a well located within the urban perimeter of the Aguachica municipality. Despite possessing a drinking water treatment system that is supplied from a surface water source, Aguachica faces water supply issues, compelling the local populace to resort to groundwater as a supplementary resource^70^.

Analyzing histograms with smoothed function fitting for total coliform analyses in each field campaign, a higher number of samples exhibiting total coliform presence was observed during the wet period (Fig. 4). The behavior associated with the occurrence of total coliforms in the analyzed samples is consistent across all three campaigns. The histograms indicate that the occurrence of total coliforms in the analyzed samples is concentrated at values below 50,000 MPN/100 mL, consistent with the analysis of central tendency measures, which reveal a mean of 13,136 MPN/100 mL and a standard deviation of 41,450 MPN/100 mL.

During the fieldwork, it was observed that the sampled wells, which are mostly located in scattered rural households, are situated near handmade pit latrines without proper hygienic protection or septic tanks and, in areas where backyard livestock production takes place. Consequently, contamination of groundwater sources by both total and fecal coliforms is expected, as evidenced by the laboratory results obtained.

**Figure 5:**
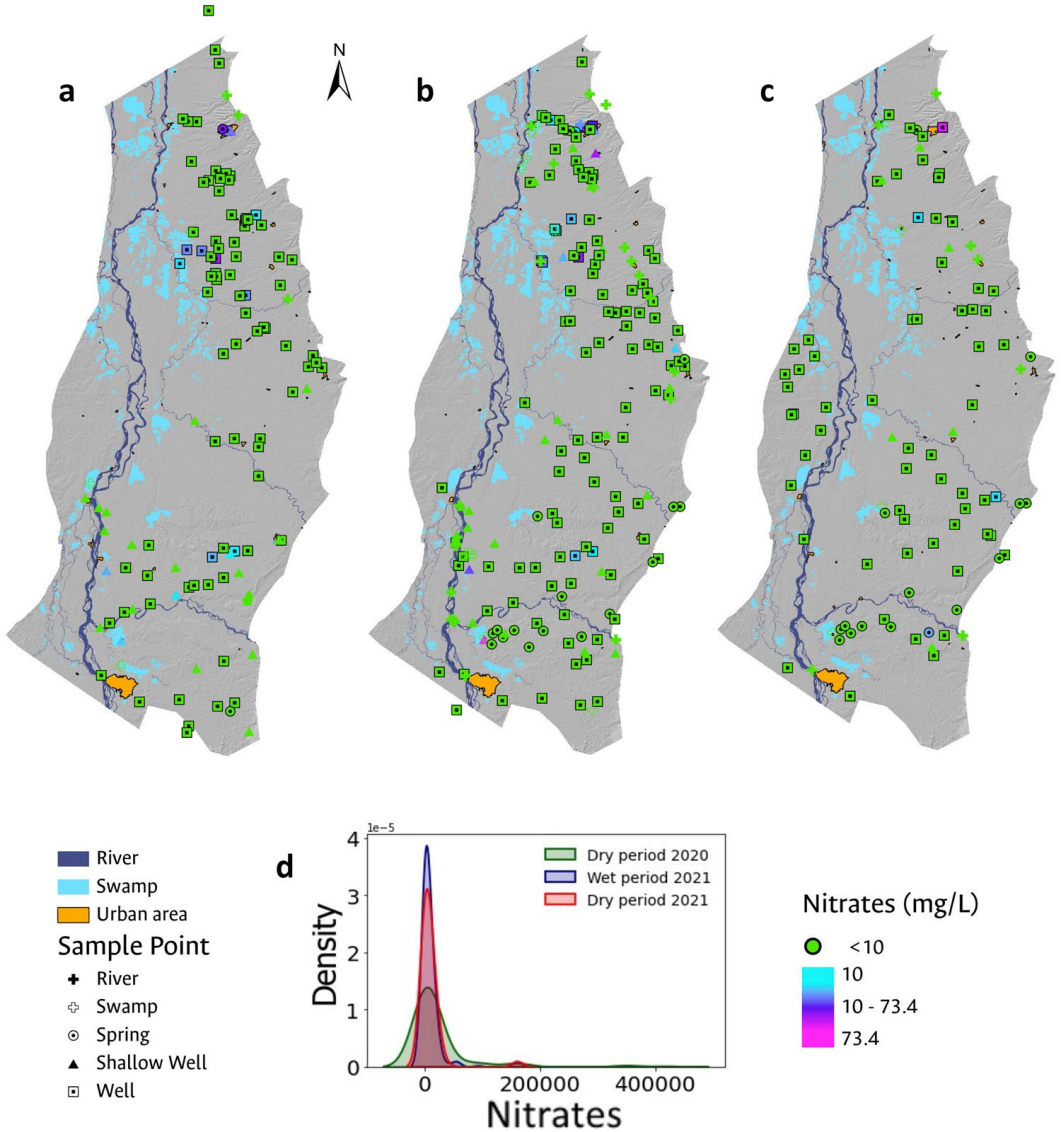
Spatial distribution of nitrate occurrence evaluated from the three conducted sampling campaigns. (**a**) Corresponds to the dry period of 2020; (**b**) to the rainy period of 2021; and (**c**) to the dry period of 2021. In (**d**), histograms with a smoothed function fit are presented for each field campaign.

Nitrates exceeded the permissible limit (10 mg/L) in more than 10% of the water samples. The spatial distribution of nitrates shown in Fig. 5 revealed higher concentrations in urban areas of Aguachica, Sabana de Torres and San Martin, probably as a result of nitrogen-rich effluent discharges counting for the lack of proper sewage treatment. High values were observed too, in specific agroindustrial zones near Puerto Wilches, where there are extensive areas of African oil palm crops, and towards Barrancabermeja, an area that was previously dedicated to crops such as rice and cotton but that nowadays is being converted into livestock^71–73^.

Histograms with smoothed function fitting for nitrate analyses indicate that nitrate occurrence is unrelated to the hydrological period during sampling Fig. 5. This is further substantiated by analyzing measures of central tendency, revealing consistent mean values of 4.3 mg/L for both dry and wet periods, with standard deviations of 9.4 mg/L and 10.7 mg/L, respectively. This finding is unexpected, considering that in Colombia, traditional farming practices often involve applying larger quantities of fertilizer directly to the soil, primarily during wet periods, to mitigate the effects of fertilizer runoff.

**Figure 6:**
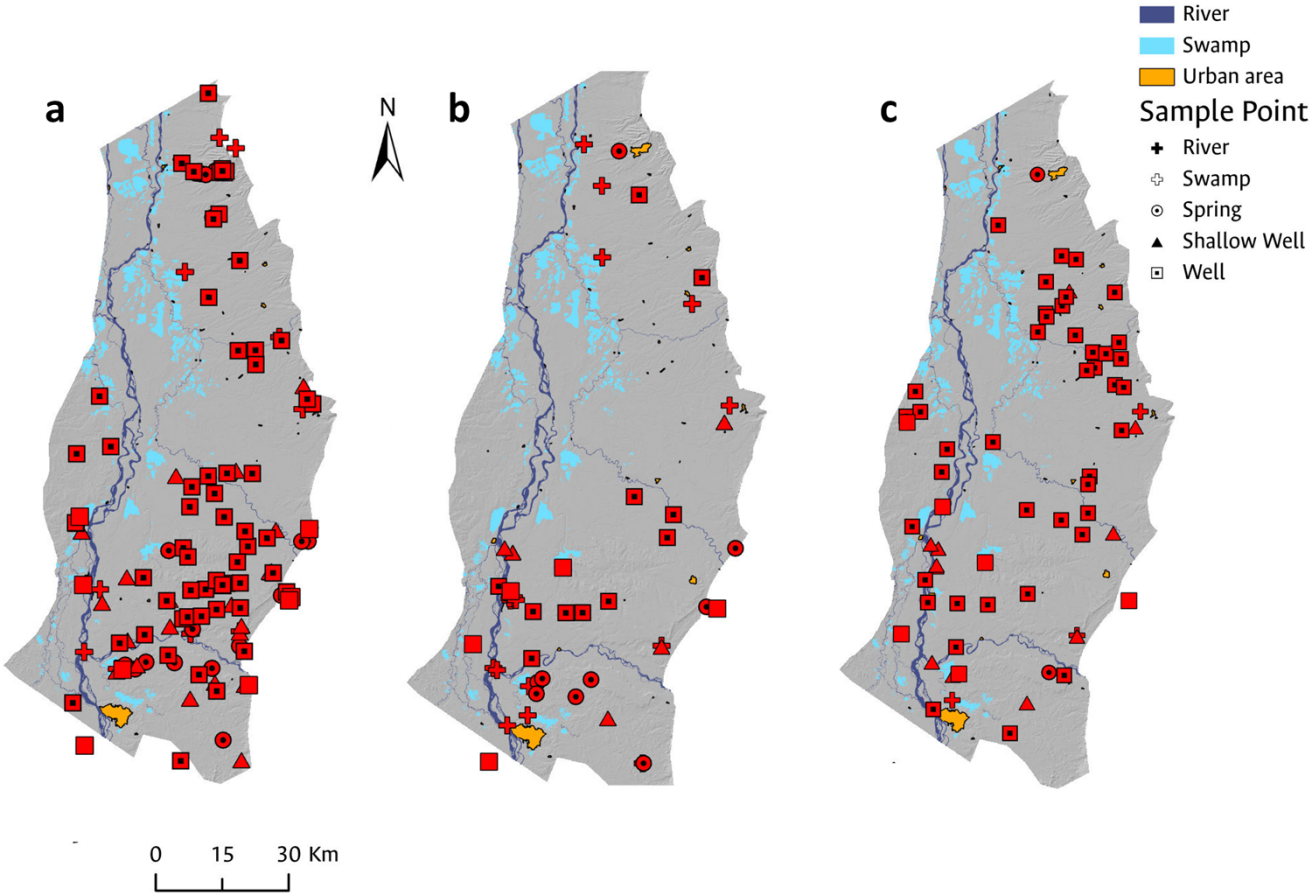
Spatial distribution of water points where analyzed samples surpass the US EPA thresholds. In (**a**) the analysis is presented for pH; in (**b**) for iron; and in (**c**) for manganese.

**Figure 7:**
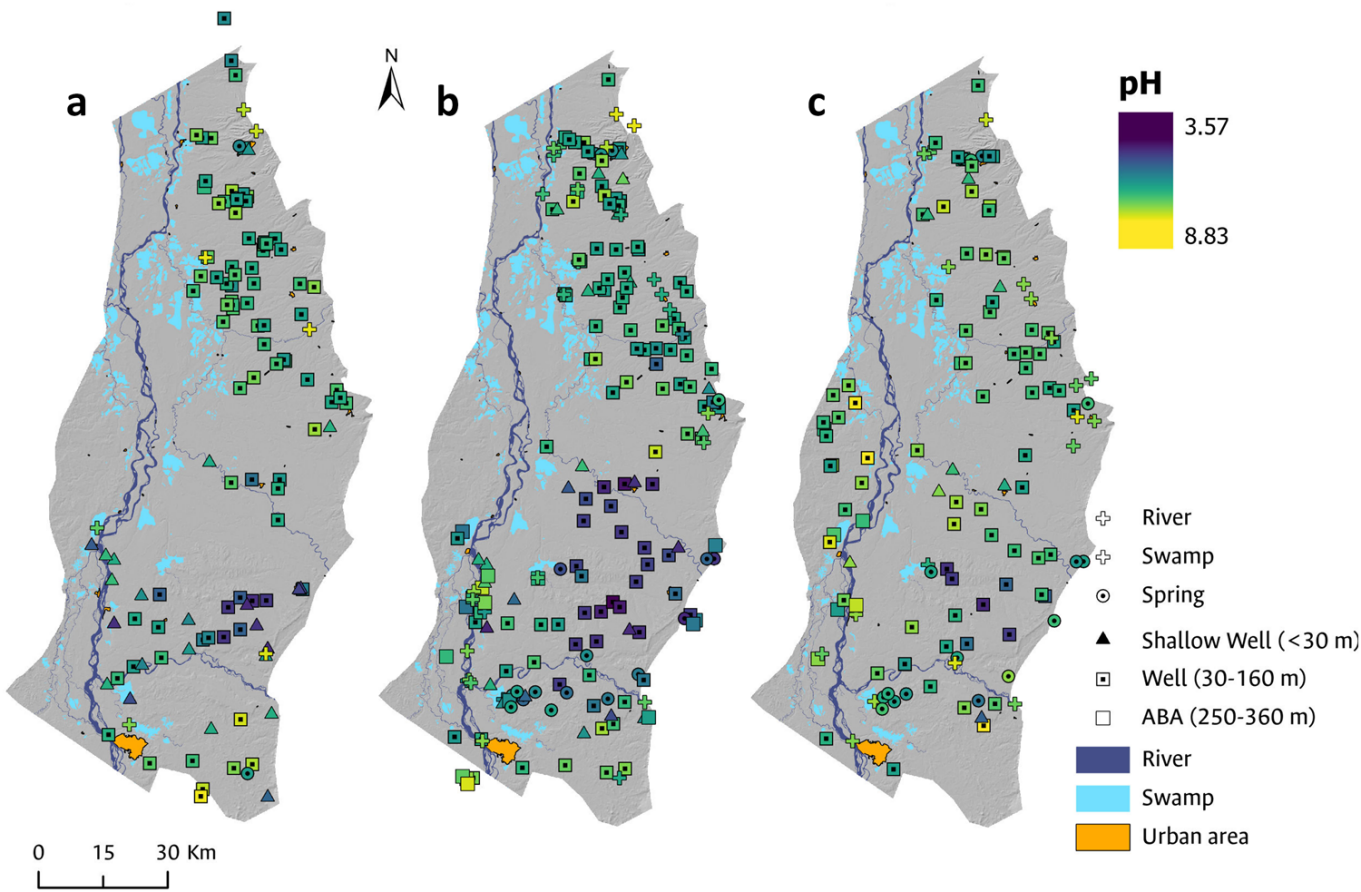
Spatial distribution of pH based on the analyzed samples. (**a**) Presents the analysis for the dry period (2020); (**b**) for the rainy period (2021); and (**c**) for the dry period (2021). pH values fluctuated between 3.57 and 8.83 in the analyzed samples.

Additionally, we conducted a spatial analysis for pH, iron and manganese, parameters that did not comply with the US EPA standards (Fig. 6). pH exceeded the permissible limit in 195 samples (40%). These samples are primarily situated between the Lebrija and Sogamoso rivers, an area predominantly characterized by African oil palm crops (Fig. 7) Iron was exceeded in 65 water samples (16.7%), mainly in the southern of the study area, possibly associated with the presence of soils rich in iron oxides. Regarding manganese, a noteworthy 21.4%, equivalent to 80 out of the total water samples were above the permissible limit. Elevated manganese concentrations are widely dispersed throughout the entire study area, implying a connection to inherent natural factors tied to the mineralogical traits of exposed geological formations, rather than anthropogenic influences^74,75^.

Finally, the presence of metals such as Ag, Pb, As, Ag and Hg is an alert for human consumption, even though those are reported in less than 3% of the water samples.

### Effect of land-use/landcover on groundwater quality

Land cover in the study area was classified into six (6) categories grouping different land use/landcover characteristics. The analysis revealed that 43.4% of the surface is covered by Grasslands, which is the predominant category and is used for extensive livestock farming. Agriculture lands occupy the second largest area with 24.9%, with African palm plantations for agro-industry and crops such as rice and corn being the most common. Forests account for 14.7% of the surface. Wetlands cover 7.9% of the area, including all marshy areas and even zones with aquatic vegetation on water bodies. Water bodies, including both lentic and lotic surface water bodies, cover 7.5% of the study area. Urban areas represent the smallest proportion of land use/land cover in the MMV, estimated to be less than 1.7% of the total surface area.

Impacts of land use and land cover on groundwater quality vary depending on the specific land categories under consideration. Agricultural lands are mainly located in the southern portion of the study area, spanning between the municipalities of Puerto Wilches and Barrancabermeja. In the northern part of the study area, grasslands predominate, primarily used for livestock farming, interspersed with scattered agricultural plots throughout the region. The precipitation distribution could potentially be linked to this behavior, given that the southern sector receives a total annual rainfall of 4000 mm, while in the northern falls below 1000 mm.

**Figure 8:**
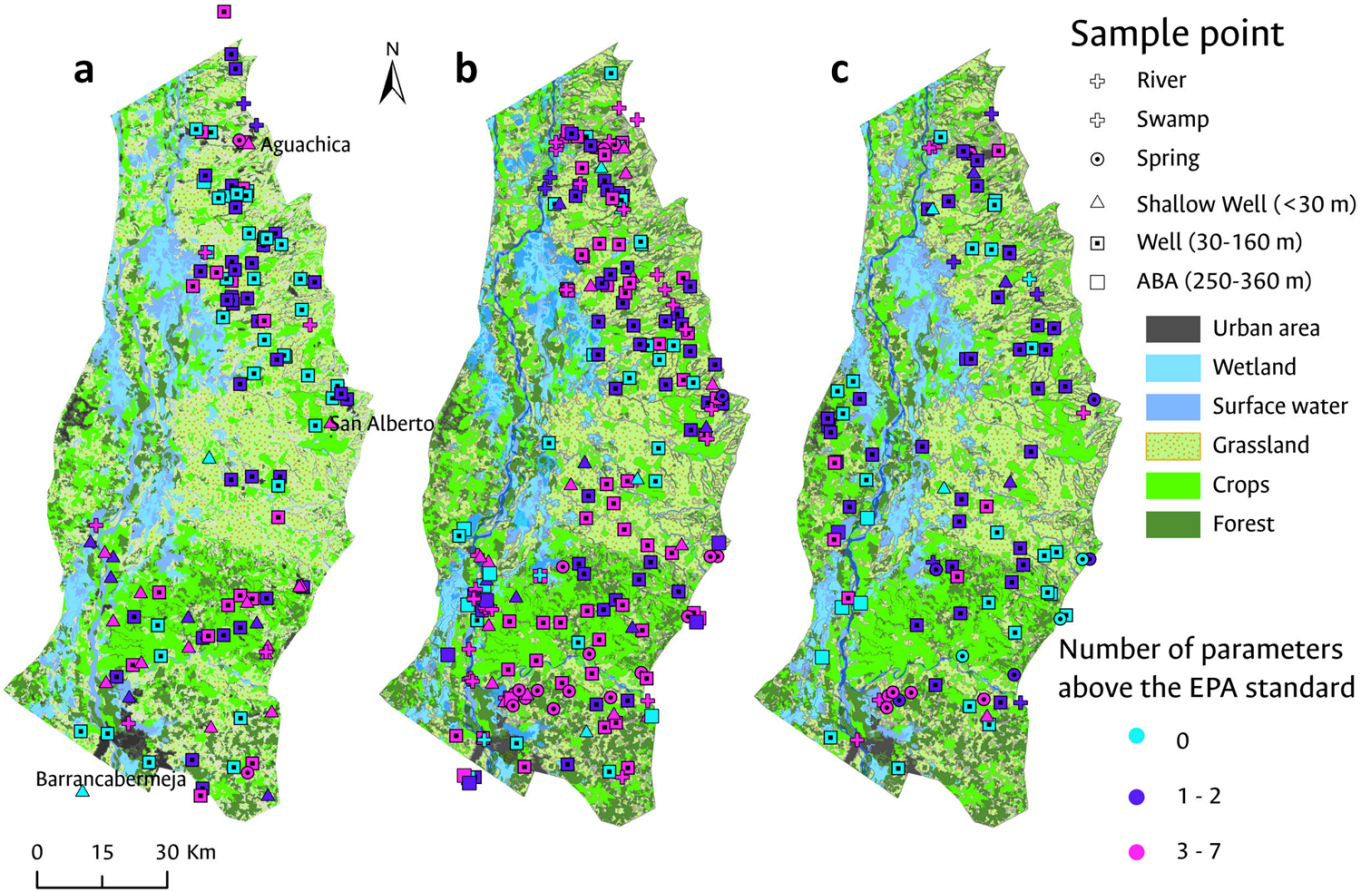
Land cover/soil use map in the MMV and the number of parameters above the US EPA standard per sample point. Land cover/use has been categorized into urban areas, wetlands, surface waters, grasslands, crops, and forests. Additionally, out of the 28 physicochemical parameters analyzed, the quantity of parameters exceeding the thresholds established by the US EPA for each sampled water point is presented. This analysis was conducted for the three campaigns: (**a**) dry period (2020); (**b**) rainy period (2021); and (**c**) dry period (2021).

Figure 8 illustrates the contrast between land use/land cover and the spatial distribution of water samples analyzed by hydrological period. The samples are categorized into three groups: (1) samples in which all the analyzed parameters are below US EPA limits, (2) those samples in which one or two parameters fail to meet the standards, and (3) those samples in which three or more parameters exceed the US EPA standards. It was observed that during the dry periods, the analyzed sampled sites complied with a greater number of parameters, especially in the northern area, characterized by grasslands. On the other hand, in the southern area, a greater number of sampled sites exhibited more than three parameters exceeding the US EPA standards in both during dry and wet periods.

Regarding the spatial distribution of nitrates in groundwater, higher concentrations were found near the main population centers lacking proper sewage systems (Aguachica, San Martín, and San Alberto), suggesting a potential association with wastewater discharges (Fig. 5). On the other hand, in the central area of the study area, characterized by scattered rural dwellings and small, low-density populations, the lowest nitrate concentrations were detected (< 5 mg/L). Temporally, the highest nitrate concentration rates occurred during the wet period, which could be linked to the increased use of pesticides, typical in Colombian agriculture practices^76^.

Lastly, shallow groundwater acidification was observed particularly concentrated between the Sogamoso River and the Lebrija River (Fig. 7). This area is characterized by African oil palm cultivation, suggesting a potential relationship between these crops, soil acidification, and consequently, groundwater acidification^77,78^.

### Assessment of human health risks

The risk of disease occurrence or health-related alterations associated with water consumption was assessed using the CDWQRI- IRCA. This index is calculated by considering the non-compliance with physicochemical and microbiological water parameters in comparison with the Colombian regulation for drinking water.

The CDWQRI-IRCA was computed for 458 water samples, spanning values from 0.0 to 79.2, with an average of 50.9, a median of 61.5, and a standard deviation of 21.1. Among the 458 samples, 386 of them, accounting for 84.28% of the total analyzed, were classified as high risk, 4.59% indicated a medium risk, 5.46% a low risk, whereas 5.68% exhibited no risk for human consumption. It is crucial to highlight that none of the samples were deemed unsuitable for consumption based on sanitary considerations.

**Figure 9:**
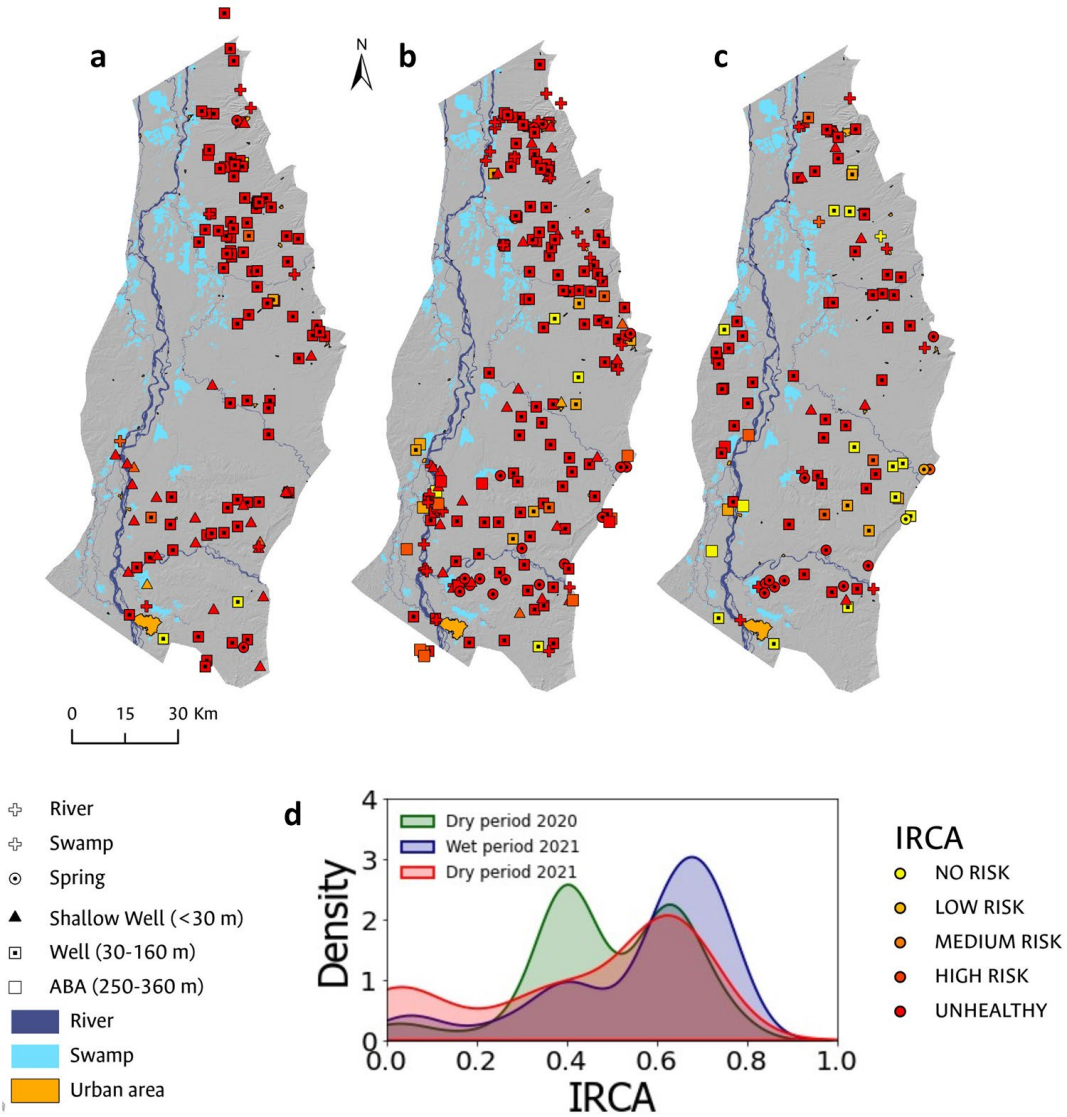
CDWQRI-IRCA values for the analyzed hydrological periods. The results of the CDWQRI-IRCA have been categorized as unhealthy, high risk, medium risk, low risk, and no risk for each conducted sampling campaign: (**a**) dry period (2020); (**b**) rainy period (2021); and (**c**) dry period (2021). Additionally, in (**d**), histograms with smoothed function fitting are presented for the results of the CDWQRI-IRCA analyses, discriminated by sampling campaign.

From CDWQRI-IRCA histograms, no significant temporal trend was observed that would allow correlating the hydrological period with the risk to human health due to the consumption of groundwater (Fig. 9). During the dry periods of 2020 and 2021, it was estimated that 90.87% and 71.84% of the sampled water points were categorized as high risk, respectively, and during the rainy period of 2020, this figure was 86.16% (Table 5).

**Table 5:** Assessment of human health risk based on CDWQRI-IRCA for the analyzed hydrological periods.

Risk classification	Dry period 2020	Wet period 2020	Dry period 2021
Unhealthy	0	0	0
High risk	119	193	74
Medium risk	5	14	6
Low risk	3	11	7
No risk	4	6	16
Total of samples	131	224	103

Spatially, in the rural area characterized by dispersed rural housing and therefore by groundwater points destined for domestic use for individual housing units, a noticeable trend was identified in the samples characterized with a high-risk level (Fig. 9). In the vicinity of the main populated centers such as Barrancabermeja, San Martín, Sabana de Torres, Aguachica, San Alberto, and San Martín, sampled sites did not exhibit a high level of risk, including a few associated with municipal water supply systems.

Therefore, a detailed analysis of the points associated with water supply systems, both municipal and rural, was conducted. Sampling took place at storage tanks to estimate the actual water quality consumed by the population. It was found that 57 points, which account for 76%, were categorized with a high-risk level for human consumption. 10 points (13%) are at a medium or low-risk level, while only 8 points (11%) pose no risks to human health.

### Direct impacts on human health

The National Institute of Health of Colombia reports two (2) deaths among children under 4 years due to ADD in 2019. One in Cantagallo and another in San Pablo on the western bank of the Magdalena River which may be related to the fact that 84% of the analyzed samples were classified as high-risk for human consumption. Unfortunately, because of the absence of health centers in rural areas and the lack of a systematic record of ADD cases, it is not possible to correlate the consumption of polluted water with the occurrence of diseases or hazards to health. In fact, it seems that people have created resistance to the DAA as not native people used to get sick when visiting the region and consuming raw water.

## Conclusions

This study assessed groundwater quality in the Middle Magdalena Valley in Colombia through the physicochemical and microbiological analysis of 28 water quality parameters. The research was conducted in three campaigns at 190 groundwater sampling points. The results showed that Ag, Se, Hg, *F*^−^, Zn, and Ba met the water quality standards proposed by the US EPA for human consumption in all analyses. Among the other parameters analyzed, both fecal and total coliforms exceeded the quality standard in a higher percentage of samples, specifically 58% and 89%, respectively.

Given that the majority of the sampled groundwater points were located in rural dwellings, where groundwater is typically used for human consumption without any treatment, and this behavior is widespread in the rural area of the Middle Magdalena Valley, an assessment of human health risks associated with the consumption of these waters was conducted. This assessment was carried out using the CDWQRI-IRCA.

The results obtained from the CDWQRI-IRCA evaluation indicate that only 5.7% of the analyzed samples pose no health risk, while 84.3% of them represent a high risk, demonstrating a correlation with the results of the total coliform analysis. As a consequence of the poor quality of groundwater, often consumed by the rural population of the MMV, two deaths of children under 4 years old due to Acute Diarrheal Disease were recorded in 2019.

The study identified that the main problem concerning water quality and its public health implications is biological contamination due to the occurrence of fecal and total coliforms at the measured groundwater points. Based on these findings, it is recommended to implement drinking water treatment systems, with an emphasis on pathogen removal and prioritizing their establishment in rural areas. Additionally, it is recommended to assess the feasibility of implementing low-cost household systems for water purification, including community aqueducts, in dispersed rural communities, with the support of organized grassroots communities. These recommendations align with the 2030 Agenda and the Sustainable Development Goals—An opportunity for Latin America and the Caribbean^79^. However, this poses a significant challenge in rural Colombia, where, by the end of 2023, only 57.8% coverage has been achieved in populated centers and scattered rural areas^80^.

While the previously mentioned solution represents a structural and largely definitive answer to the problem, alternative measures are needed to address this issue. Among these, the need to ensure protection systems in wells to be constructed, such as sanitary seals, stands out; avoiding the construction of wells in the vicinity of handmade pit latrines or areas of wastewater discharge, especially if they are upstream of the wells to be built for human supply; abandoning wells that are within 50 m of wastewater discharge points or handmade pit latrines^81^; intensifying governmental efforts in constructing effective wastewater treatment systems, both in urban and rural areas; ensure compliance with national regulations for the discharge of industrial wastewater by constructing industrial wastewater treatment systems in agro-industrial companies within the territory; establish systematic groundwater monitoring plans at both national and regional levels, incorporating participative monitoring processes and promote water governance; and implement environmental education campaigns and processes designed to enhance awareness and understanding of water conservation, utilization, and management. These initiatives should be aligned with a National Environmental Education Policy and thus be integrated into both national and regional environmental education programs to ensure the sustainability of the actions undertaken.

This research represents an effort to assess the quality of groundwater and understand its potential public health impacts. However, there is a need for greater understanding of the possible impacts that climate change could induce, particularly regarding changes in land use, anthropogenic pressures on groundwater, and aquifer recharge, which are intrinsically related to alterations in precipitation patterns. Therefore, it is proposed that long-term groundwater quality monitoring strategies be implemented and that future studies be conducted to examine the effects of climate change on groundwater quality in the MMV at a regional scale.

### Supplementary Information

Supplementary Information 1.

Supplementary Information 2.

Supplementary Information 3.

Supplementary Information 4.

## Data Availability

The datasets used and analysed during the current study are available from the first author on reasonable request.
